# Prevalence of Hepatitis E Virus Infection Among Pregnant Women in Tunisia: Findings from a Large Cohort Study

**DOI:** 10.3390/pathogens15050549

**Published:** 2026-05-19

**Authors:** Kaouther Ayouni, Mariem Gdoura, Rania Allègue, Majdi Ben Ameur, Henda Touzi, Nesrine Abderahmane, Khaoula Magdoud, Hiba Mkadmi, Rim Ben Hmid, Henda Triki, Anissa Chouikha

**Affiliations:** 1 Laboratory of Clinical Virology, WHO Reference Laboratory for Poliomyelitis and Measles in the Eastern Mediterranean Region, Pasteur Institute of Tunis, University of Tunis El Manar, 13 Place Pasteur, BP 74, Tunis 1002, Tunisia; mariemgdoura@gmail.com (M.G.); raniaallegue95@gmail.com (R.A.); majdibenameur9@gmail.com (M.B.A.); touzihenda@yahoo.fr (H.T.); nesrine.abde123@gmail.com (N.A.); henda.triki@pasteur.tn (H.T.); chouikhaanissa@gmail.com (A.C.); 2 Research Laboratory, “Virus, Vector and Host: One Health Approach and Technological Innovation for a Better Health” (LR20IPT02), Pasteur Institute of Tunis, University of Tunis El Manar, 13 Place Pasteur, BP 74, Tunis 1002, Tunisia; 3 Faculty of Sciences of Tunis, University of Tunis El Manar, Campus Universitaire El Manar, Tunis 2092, Tunisia; 4 Clinical Investigation Center (CIC2016IPT02), Institut Pasteur de Tunis, University of Tunis El Manar, 13 Place Pasteur, Tunis 1002, Tunisia; 5 Faculty of Pharmacy of Monastir, University of Monastir, Monastir 5000, Tunisia; 6 Department of Gynecology Emergency, Center of Maternity and Neonatology, Boulevard 9 Avril, Tunis 1006, Tunisia; magdoudkhaoula@gmail.com (K.M.); hibamkadmi03@gmail.com (H.M.); rimbenhmid@yahoo.fr (R.B.H.); 7 Faculty of Medicine of Tunis, University of Tunis El Manar, 15 Rue Djebel Lakhdhar, La Rabta, Tunis 1007, Tunisia

**Keywords:** hepatitis E virus, hepatitis E infection, prevalence, serology, Tunisia, pregnancy

## Abstract

Hepatitis E is a liver inflammation caused by the hepatitis E virus (HEV). In pregnant women, the infection significantly increases the risk of acute liver failure, fetal loss, and maternal death. According to the World Health Organization, infection by HEV during the third trimester of pregnancy may increase the risk of maternal mortality in 20–25% of cases. In Tunisia, little is known about HEV infection and its outcome, especially in pregnant women. This study aims to evaluate the prevalence of HEV infection in a large cohort of pregnant women in Tunisia. A total of 891 women who attended the Centre of Maternity and Neonatology of Tunis during 2021–2023 were included. Serum samples were screened to detect HEV-antibodies and RNA using commercial ELISA tests and molecular assays, respectively. Statistical analyses were conducted using SPSS 21.0 software and the EPISTAT package version 7.2.6. Seroprevalence of HEV infection was 3.82%, based on the detection of anti-HEV IgG. The distribution of the seroprevalence according to age was statistically significant (*p* < 0.05), showing a higher seroprevalence among women over 30 years. Among the 51 women with composite outcomes, viral RNA was detected in one case by real-time RT-PCR. Our findings indicate a low HEV prevalence among pregnant women in Tunisia. Expanding the study to other cohorts and to environmental surveillance would improve understanding of HEV burden in Tunisia and support hepatitis elimination efforts.

## 1. Introduction

Hepatitis E is a liver inflammation caused by the hepatitis E virus (HEV). HEV is a positive-sense, single-stranded RNA virus belonging to the family of *Hepeviridae* and the genus *Orthohepevirus*. It is classified into a single serotype and eight genotypes [1,2,3,4]. According to the World Health Organization (WHO), in 2021, HEV infection was responsible for 3450 deaths worldwide and approximately 19.47 million cases of acute hepatitis E globally [5]. The prevalence of HEV varies across geographic regions, migrant populations, clinical cohorts, and occupational groups, reflecting both waterborne and zoonotic transmission patterns. Two broad epidemiological patterns are recognized: high endemicity in many developing countries, caused by waterborne HEV genotypes 1 and 2 and associated outbreaks, and sporadic zoonotic cases in developed countries mainly induced by HEV genotypes 3 and 4 [1,2,3,4].

Although HEV infection is usually acute and self-limiting in the general population, certain groups, including pregnant women, immunocompromised individuals (e.g., hemodialysis, HIV-positive individuals, and patients receiving immunosuppressive therapies), workers exposed to animals or wastewater, and people living in endemic areas with poor sanitation, are considered high-risk populations for HEV [6,7,8,9,10,11]. HEV infection during pregnancy is associated with severe outcomes, including fetal loss, fulminant hepatic failure (FHF), and maternal mortality rates of up to 30% [12]. FHF is considered the leading cause of the high mortality rate observed among pregnant women, especially in the third trimester [13,14,15]. Anti-HEV IgG seroprevalence in pregnant women shows substantial regional variation, ranging from approximately 6% in low-endemic areas to over 45% in some Middle Eastern countries. Global estimates suggest a pooled prevalence of 16.5%, with Africa at 29%, Southeast Asia around 18%, and the highest in the Middle East at approximately 48% [6,7,8].

In Tunisia, limited data are available on HEV infection such as its prevalence and circulating genotypes. According to the few studies conducted on HEV infection in Tunisia, its prevalence was estimated from 4.3% to 5.4% in the general population, 4.5 to 5.4% in blood donors, 3 to 19.5% in patients with acute hepatitis, 7.5% in Hemodialyzed patients, 10.2% in hemophiliacs, 28.9% in poly-transfused persons, 2% in wastewater and from 5.1 to 12.1% in pregnant women [16,17,18,19,20,21,22,23]. Variations in study periods, sample sizes, and socioeconomic characteristics of the populations may influence the observed differences in HEV seroprevalence. In fact, most of these studies, especially those in pregnant women, were conducted on relatively limited cohorts and using different assays. This highlights the need for up-to-date, well-designed studies to provide more accurate estimates of HEV seroprevalence and a better understanding of its epidemiological dynamics. The present study was conducted to assess HEV prevalence in a large, recent cohort and to provide updated estimates among pregnant women in Tunisia.

## 2. Materials and Methods

### 2.1. Enrollment Site and Study Population

Pregnant women were recruited between 2021 and 2023 from the Neonatal Resuscitation Department of the Centre of Maternity and Neonatology of Tunis (CMNT), Tunisia, as part of the WHO-funded Preg-Cov project (WHO reference: 2023/1329040-0) [24]. It is the leading reference centre in Tunisia for advanced obstetric and neonatal care, comprising 4 gynecology–obstetrics departments, an emergency department, an outpatient department, an adult intensive care department and a neonatal medicine and resuscitation department with an average of 80 newborns hospitalized per day. The annual number of deliveries is estimated at 10,000, with more than 3000 neonatal hospitalizations. In addition, the CMNT covers the entire region of Tunis (the capital and largest city) and the north-west of the country, as well as receives in utero transfers from all over Tunisia, including the private sector. Pregnant women at any gestational age attending antenatal care or other obstetric services at the CMNT were consecutively recruited, regardless of underlying comorbidities. Pregnant women who were unavailable for follow-up (i.e., planning delivery in another city or facility) and pregnant women who were incapable of providing informed consent or assent were excluded. Peripheral blood was drawn from each participant and a biobank was established, with serum samples stored at −20 °C.

### 2.2. Clinical Specimens

A total of 891 serum samples were included. All samples were maintained in the biobank of the Clinical Virology Laboratory at the Institut Pasteur de Tunis under strict biosafety and biosecurity standards. Each sample was accompanied by a questionnaire, and a database was compiled with socio-demographic and clinical information. Data related to any etiology, such as HIV-infection, presence of chronic or immune-related diseases, were collected.

### 2.3. Serological Tests

Screening of the anti-HEV IgM and anti-HEV IgG antibodies was conducted using the Abia HEV IgM and Abia HEV IgG ELISA kits (AB Diagnostic Systems GmbH, Berlin-Adlershof, Germany), respectively. Positive IgM results were confirmed using another ELISA test, the WANTAI HEV-IgM ELISA kit (Beijing Wantai Biological Pharmacy Enterprise Co., Ltd., Beijing, China). The serological results were interpreted according to the manufacturer’s instructions. For WANTAI HEV-IgM ELISA, the cut-off value was calculated as the mean Optical Density (OD) of negative controls plus 0.26, and results were expressed as Ratio = Absorbance/Cut-off (A/C.O). Samples were considered positive if the ratio was ≥1 and borderline if it was between 0.9 and 1.1. For the Abia HEV IgM and IgG ELISA kits, the cut-off was defined as the mean OD of negative controls plus 0.200, with samples considered positive when OD ≥ the cut-off.

### 2.4. Molecular Assays

The RNA detection was performed using nested and real-time RT-PCR on samples that were positive for anti-HEV IgM by AB Diagnostic Systems GmbH, Germany kit, as well as on samples of women presenting composite outcomes. Viral RNA was extracted from 140 µL of serum using the QIAamp viral RNA mini-Kit (QIAGEN, Hilden, Germany) following the manufacturer’s instructions. A synthetic positive control was used in this study, consisting of a plasmid containing the target sequence corresponding to the genomic regions amplified by both the nested PCR and real-time RT-PCR assays.

A Taq-Man real-time RT-PCR targeting 70 bp of the ORF2/ORF3 region was performed for rapid detection of HEV RNA using previously published primers: forward primer: 5′-GGTGGTTTCTGGGGTGAC-3′, reverse primer: 5′-AGGGGTTGGTTGGATGAA-3′, and the probe: 5′-FAM-TGATTCTCAGCCCTTCGC-TAMRA-3′ and the SuperScript III Platinum One-Step qRT-PCR Kit (Invitrogen, Thermo Fisher Scientific, Waltham, MA, USA) [25]. The reaction mixture contained primers (400 nM), probes (400 nM), 12.5 µL of 2X master mix, 1 unit of SuperScript III Platinum One-Step enzyme, 8 µL of RNA, and nuclease-free water qsp 50 µL.

One-step real-time RT-PCR was performed with LineGene 9600 real-time RT-PCR Detection System (Hangzhou Bioer Technology Co., Ltd., Hangzhou, China). Reverse transcription was carried out at 50 °C for 20 min, followed by a step of denaturation at 95 °C for 3 min, 45 cycles of denaturation at 95 °C for 15 s, annealing at 57 °C for 5 s and elongation at 60 °C for 30 s. The sensitivity of the real-time RT-PCR was estimated by testing a serial dilution of the synthetic positive control with known copy numbers.

Nested PCR was performed to detect 457 bp of the ORF2 region and 137 bp of the ORF2/ORF3 overlapping region of HEV RNA. Reverse transcription and amplification were carried out using previously published primers [26,27]. The primers are listed in Table 1.

For both regions, the cDNA was synthesized using 10 µL of RNA and 0.5 µM of reverse primers. The amplification was then carried out with 10 µL of cDNA and 0.2 µM of each of the reverse and forward primers at 94 °C for 5 min, followed by 35 cycles (30 s at 94 °C, 30 s at 58 °C, 1 min at 72 °C) and a final elongation at 72 °C for 7 min. Nested PCR was carried out with 5 µL of the product of the first PCR and 0.2 µM of inner primers, following the same thermal conditions. The limit of detection of the nested PCR was estimated by testing the same serial dilution of the synthetic positive control.

### 2.5. Statistical Analysis

Results were analyzed using SPSS 21.0 software package (SPSS; IBM, Armonk, NY, USA) to assess the association of anti-HEV status with potential risk factors. Statistical analysis was performed using Pearson’s chi-square test or Fisher’s exact test when appropriate. Yates’ continuity correction was applied for 2 × 2 tables with small expected frequencies. Analyses were conducted using available data for each variable; missing data were not imputed, and denominators therefore varied between analyses. Seroprevalence estimates were reported with 95% confidence intervals calculated using the Wilson score method. Odds ratios (ORs) and 95% confidence intervals (CIs) were calculated using the EPISTAT statistical package version 7.2.6 (Centers for Disease Control and Prevention, Atlanta, GA, USA). A *p*-value < 0.05 was considered statistically significant.

## 3. Results

### 3.1. Socio-Demographic Characteristics of the Study Population

The age of our study population ranged from 18 to 46 years (mean: 32 ± 5.4 years). The gestational age at the time of enrollment ranged from 1 to 42 weeks. Of the 891 participants, 68.36% of the pregnant women were in the third trimester of pregnancy. Two women were HIV-positive and seven had immune dysfunction (multiple sclerosis (*n* = 1), asthma (*n* = 1), allergy (*n* = 4), and Crohn’s disease (*n* = 1). Out of the 786 cases for which delivery data were accessible, 735 (93.51%) resulted in live births, while 51 (6.49% of cases) had a composite outcome: 9 (1.15%) stillbirths, 22 (2.80%) spontaneous abortions, and 20 (2.54%) therapeutic abortions.

The distribution of participants according to geographic location or area is characterized by a significant prevalence in the northern part of Tunisia, accounting for 75.92% (631 cases). Among the participants, 48.55% (368/758) had less than a secondary education, 32.32% of participants (245/758) had completed secondary education, while 19.13% (145/758) had attained a university education or higher.

### 3.2. HEV Seroprevalence

Anti-HEV IgG antibodies were detected in 34 of 891 samples, corresponding to a seroprevalence of 3.82% (95% CI: 2.56–5.08%). No sample was positive for anti-HEV IgM antibodies. Among the 891 sera tested, 87 samples (9.76%) yielded positive IgMs using the ABIA ELISA assay (tested twice as recommended by the manufacturer); however, all these samples were subsequently found to be negative when re-tested with the WANTAI HEV ELISA kit. Referring to the manufacturer’s data, the Wantai IgM assay showed no interference from rheumatoid factors up to 2000 U/mL, supporting its high specificity.

The distribution of the HEV IgG seroprevalence according to age group was statistically significant (Table 2). The seroprevalence increased with age, from 2.1% in women ≤ 30 years to 4.8% in those >30 years (*p* = 0.038, χ^2^ = 4.54, OR = 2.41). The distribution of anti-HEV IgG seroprevalence was similar in urban and semi-urban areas (3.6% vs. 3.7%, *p* = 0.945, χ^2^ = 0.005, OR = 0.945) (Table 2). HEV IgG seroprevalence was higher among pregnant women with a university education (4.8%) and those with primary/secondary education (3.2%) compared to those with a lower level of education (0.9%); however, this difference was not statistically significant (*p* = 0.218) (Table 2).

### 3.3. Molecular Results 

#### Optimization with a Synthetic Positive Control

**Figure 1:**
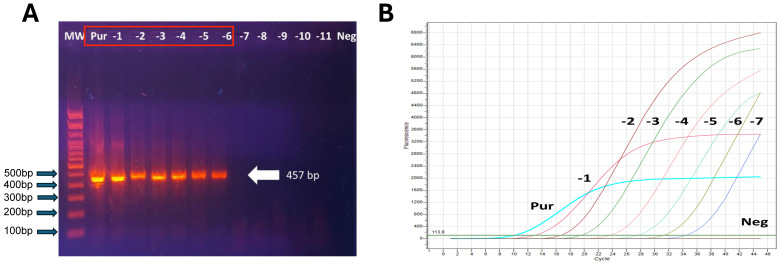
Amplification and detection of the hepatitis E virus ORF2 fragment by conventional PCR and real-time RT-PCR: (**A**) Agarose gel electrophoresis of PCR products targeting the ORF2 region of the hepatitis E virus (HEV) genome. Lanes correspond to serial dilutions of the synthetic positive control. A specific amplicon of approximately 457 bp is observed, confirming the specificity of the assay. The DNA molecular weight marker (100–500 bp) is shown on the left; (**B**) Real-time RT-PCR amplification curves obtained from serial dilutions of the synthetic positive control (Pur), demonstrating the analytical sensitivity and dynamic range of the assay. No amplification is observed in the negative control (Neg) (see Figure S1 for the original Western blotting image).

The optimization step was carried out using a synthetic positive control. HEV RNA of the synthetic positive control was detected using nested PCR targeting 457 bp of the ORF2 region and 137 bp of the ORF2/ORF3 overlapping region. The limit of detection was around 8 × 10^3^ copies/µL (Figure 1A). Results showed that the detection limit of the real-time RT-PCR assay was around 8 × 10^2^ copies/µL (Figure 1B).

The 87 sera that tested IgM-positive by the ABIA ELISA assay and IgM-negative by the WANTAI HEV ELISA kit were screened for HEV RNA by real-time RT-PCR and nested PCR. HEV RNA was not detected in any of the analyzed samples.

Interestingly, among the 51 women with composite outcomes who tested negative for both anti-HEV IgM and anti-HEV IgG, viral RNA was detected in one case by real-time RT-PCR, with a high cycle threshold (Ct = 36.8), suggesting a low viral load. However, viral RNA could not be detected using end-point RT-PCR. The case corresponded to a 30-year-old pregnant woman who experienced a spontaneous abortion at 12 weeks of gestation (end of 1st trimester). In this case, the pregnancy was confirmed to be non-ectopic.

## 4. Discussion

Our study assessed the prevalence of HEV among a cohort of 891 pregnant women in Tunisia. Anti-HEV IgG antibodies were detected in 34 participants (3.8%), indicating prior exposure to the virus in a subset of the cohort. There are no official quantitative guidelines from the WHO defining levels of hepatitis E endemicity based on IgG seroprevalence. However, according to published studies, hepatitis E prevalence in Tunisia can be considered as indicative of low endemicity [28].

Compared to the published data from other countries, the seroprevalence of HEV among pregnant women in our study was similar to that found in Italy (3.4%) but lower than that reported in Iran (6.2%), China (6.6%), Cameroon (9.0%), Nigeria (9.9%), Sudan (12.5%), Gabon (14.1%), and Egypt (58.6%) [29,30,31,32,33,34,35,36]. Differences in seroprevalence may be attributed to variations in living conditions, diagnostic approaches, and epidemiological contexts, as well as to the predominant transmission route in endemic countries where infection occurs mainly through contaminated water [6,7,8].

The seroprevalence observed in the current study among pregnant women was lower than rates previously reported in Tunisia. Hannachi et al. documented the highest prevalence, with 12.1% of pregnant women testing positive in 2006, whereas Neffati et al. reported a prevalence of 5.1% in 2014–2015 [22,23]. Discrepancies between studies may be attributed to several factors, including differences in study periods, sample size, socioeconomic profiles, and the commercial assays used for antibody detection. In fact, the study conducted by Neffati et al. involved a smaller cohort of 216 asymptomatic pregnant women from southern Tunisia [22]. Beyond sample size differences, the higher seroprevalence reported by Hannachi et al. may be explained by the characteristics of their study population: among the 404 pregnant women screened for HEV antibodies, 33.2% lived in rural areas in central-eastern Tunisia and 16% had a low socioeconomic status [23]. Moreover, differences in ELISA kits used to estimate anti-HEV IgG seroprevalence in Tunisia should be considered a potential confounding factor. Earlier studies employed the Globe Diagnostics ELISA, which is less sensitive and may fail to detect low antibody levels compared with the Wantai assay, known for its higher sensitivity and specificity [16,20]. More recent studies using the Wantai kit tend to report slightly higher seroprevalence rates, making direct comparisons challenging [20,22]. Therefore, observed variations in seroprevalence may partly reflect differences in IgG assay performance over time rather than true epidemiological changes.

Compared with nationwide surveys conducted in large cohorts from the general population, our prevalence estimate is slightly lower than reported (4.3% and 5.4%) for anti-HEV IgG [16,20]. Our findings, derived from a relatively large and recently established cohort, provide solid and contemporary evidence on HEV seroprevalence in Tunisia. These results may reflect the substantial improvements in hygiene and sanitation achieved nationally. Tunisia has strengthened wastewater treatment infrastructure and expanded access to safe, regulated drinking water. As the hepatitis E virus is mainly transmitted through contaminated water, these advances likely contribute to the lower prevalence observed. Moreover, consistent with local dietary practices, meat is generally consumed well-cooked, thereby limiting the risk of infection [5]. Importantly, this study establishes an updated and reliable baseline for HEV epidemiology in Tunisia. Although the Centre of Maternity and Neonatology of Tunis serves as a major national referral centre with a high patient number and wide geographic coverage, including referrals from across the country, recruitment from this single tertiary care setting is likely to overrepresent complicated or high-risk pregnancies. Consequently, the study population may not fully reflect the general pregnant population, and caution is warranted when extrapolating these findings to the national level. Nevertheless, the persisting seroprevalence remains noteworthy and highlights the need for sustained surveillance. Extending investigations to other population groups and integrating environmental surveillance approaches within the frame of the one-health concept would enable a more precise assessment of the HEV burden in Tunisia and further support national and global hepatitis elimination efforts.

In the present study, a statistically significant association was observed between HEV infection and age, with higher seroprevalence detected among women older than 30 years. This finding aligns with previous Tunisian studies conducted in pregnant women and in the general population, which have shown that the risk of HEV infection increases with advancing age, reflecting cumulative exposure over time [16,20,23]. Age greater than 30 years has been identified as the strongest independent predictor of HEV seropositivity, with seroprevalence increasing from approximately 2.2% in younger adults to about 8% in older adults (adjusted odds ratio ≈ 3.0) [20]. Houcine et al. reported that HEV seroprevalence was also associated with residence in rural or low-income regions, high-density urban areas and low educational level [20]. However, in our study, no statistically significant association was observed between HEV infection and either educational level or area of residence. This difference may be explained by the fact that our study population included only urban and semi-urban settings. Furthermore, despite lower educational levels in some participants, ongoing awareness programs emphasizing personal and collective hygiene, as well as the use of safe drinking water, may have reduced the risk of HEV infection.

In the present study, no HEV IgM–positive cases were detected, indicating the absence of confirmed recent HEV infection. In previous studies conducted among pregnant women in Tunisia, the reported prevalence of anti-HEV IgM ranged from 0.97% to 1.4% [23,24]. Our findings may be explained by the absence of clinical symptoms consistent with probable hepatitis infection in all enrolled women. Two different commercial ELISA kits were used, one of which yielded a relatively high IgM positivity rate of approximately 10% and the other kit found no positive sample. This discrepancy may be attributed to potential cross-reactivity with rheumatoid factors, which are frequently present in pregnant women. In fact, the Wantai ELISA kit, used in our study for anti-IgM confirmation, has been reported to demonstrate higher sensitivity and specificity compared with other immunoassays [37,38]. Several studies reported that false positive IgM results may occur and that there is a need for confirmation using a second serological or molecular assay [22,39,40]. In an Italian acute hepatitis cohort, four samples initially positive by Bioelisa IgM were negative on confirmatory testing with Wantai and MP-Diagnostics assays, with one case influenced by concurrent CMV infection [39]. In pregnant cohorts, especially where HEV prevalence is low, IgM ELISAs may generate false positive results [22,40]. Three weakly reactive anti-HEV IgM results without detectable HEV RNA were reported among asymptomatic pregnant women in a southern Tunisia cohort [22], suggesting likely false positives in pregnancy. Two IgM-reactive samples in an Argentinian pregnant cohort were also RNA-negative, raising similar uncertainty about serology-only diagnosis [40]. These observations highlight the need for cautious interpretation of IgM results and the importance of confirmatory testing. Therefore, confirmation of IgM-positive results using molecular techniques or alternative ELISA assays is essential.

Molecular testing revealed that one serum sample was positive for HEV RNA by real-time RT-PCR, suggesting a possible active HEV infection in a pregnant woman who experienced a spontaneous abortion at the end of the first trimester. This sample was positive only by real-time RT-PCR (Ct = 36.8) and negative by nested PCR, suggesting a low viral load. While HEV infection has been associated with adverse pregnancy outcomes, including miscarriage, the present observation does not allow a causal relationship to be established.

Several large cohort and outbreak reports consistently describe spontaneous abortion, intra-uterine fetal death, and stillbirth as frequent complications when infection occurs in the third trimester. To the best of our knowledge, no specific data on the relationship between first-trimester abortion and HEV infection have been reported. However, in the present study, a causal relationship between HEV infection and abortion cannot be excluded or confirmed and therefore remains hypothetical; nevertheless, HEV infection may be considered a possible contributing factor, particularly given that the pregnancy was confirmed to be non-ectopic.

This study has some limitations that should be acknowledged. As the cohort was initially established for a COVID-19 study, some variables relevant to HEV infection were not specifically collected. First, occupational data were obtained through a structured questionnaire; however, the classification applied does not enable stratification according to exposure risk or socioeconomic status. Second, no specific data were collected on exposure to animals, which restricts the assessment of this potential risk factor and may have influenced the analysis of associations with HEV infection. Third, the use of serum samples may not be the optimal specimen type for HEV molecular detection due to its fecal–oral transmission route, potentially affecting detection sensitivity.

## 5. Conclusions

In conclusion, the present study provides valuable insights into the prevalence of HEV among pregnant women in Tunisia. It establishes robust molecular tools for HEV detection that can be applied in future epidemiological investigations and for the diagnosis of hepatitis of unknown etiology. Our findings indicate a low HEV seroprevalence among pregnant women in Tunisia but highlight the need for further research to accurately assess national prevalence and support hepatitis elimination strategies. Expanding studies to larger cohorts, particularly among high-risk populations, would enhance understanding of the epidemiology of HEV infection in Tunisia. In addition, further investigations based on molecular surveillance, using both human and environmental samples, are needed to better clarify local transmission dynamics.

## Figures and Tables

**Table 1:** Primers used for nested RT-PCR for the detection of hepatitis E infection.

Target Region	Primer Sequence (5′-3′)	Product Length (bp)	Primer Position *
ORF2/ORF3	GCRGTGGTTTCTGGGGTGAC	164	5259–5278
	CTGGGMYTGGTCDCGCCAAG		5422–5403
	GYTGATTCTCAGCCCTTCGC	137	5282–5301
	GMYTGGTCDCGCCAAGHGGA		5418–5399
ORF2	CAAGGHTGGCGYTCKGTTGAGAC	506	5912–5934
	CCCTTRTCCTGCTGAGCRTTCTC		6417–6395
	GYTCKGTTGAGACCWCBGGBGT	457	5922–5943
	TTMACWGTCRGCTCGCCATTGGC		6378–6356

* Primer positions are based on GenBank sequence accession no. M73218.

**Table 2:** Distributions of anti-HEV antibodies in pregnant women based on demographic characteristics.

Variable	N	IgG Positive n (%)	95% CI (Wilson)	Test (df)	*p*-Value	OR (95% CI)
**Age**						
≤30	339	7 (2.1%)	1.0–4.2	χ^2^ = 4.31 (1)	0.038	0.41 (0.17–0.97)
>30	497	24 (4.8%)	3.3–7.1			2.41 (1.02–5.65)
**Residence area**						
Urban	503	18 (3.6%)	2.3–5.6	χ^2^ = 0.005 (1)	0.945	0.97 (0.46–2.05)
Semi-urban	327	12 (3.7%)	2.1–6.3			1.03 (0.49–2.16)
**Education level**						
Illiteracy/Less than Primary	107	1 (0.9%)	0.2–5.1	Fisher’s exact	0.218	0.26 (0.03–1.93)
Primary/Secondary	506	16 (3.2%)	2.0–5.1			0.99 (0.42–2.36)
University	145	7 (4.8%)	2.4–9.6			1.78 (0.72–4.37)

Significant *p*-value (<0.05). OR: Odds ratio; 95% IC: 95% confidence interval; df: degrees of freedom. The number of observations varies across variables due to missing data. Percentages were calculated using available data for each subgroup. Confidence intervals for seroprevalence were calculated using the Wilson method. Group comparisons were performed using Pearson’s chi-square test or Fisher’s exact test when appropriate. Yates’ continuity correction was applied for 2 × 2 tables when required.

## Data Availability

The data presented in this study are available upon request from the corresponding author due to ethical restrictions.

## Supplementary Materials

The following supporting information can be downloaded at: https://www.mdpi.com/article/10.3390/pathogens15050549/s1, Figure S1: The original Western blotting image of Figure 1A.

## Abbreviations

The following abbreviations are used in this manuscript:

HEV	Hepatitis E virus
WHO	World Health Organization
FHF	Fulminant hepatic failure
CMNT	Centre of Maternity and Neonatology of Tunis
OR	Odds ratio
95% IC	95% confidence interval

## References

[1] Zhou J.H., Li X.R., Lan X., Han S.Y., Wang Y.N., Hu Y., Pan Q. (2019). The genetic divergences of codon usage shed new lights on transmission of hepatitis E virus from swine to human. Infect. Genet. Evol..

[2] Zhou X., de Man R.A., de Knegt R.J., Metselaar H.J., Peppelenbosch M.P., Pan Q. (2013). Epidemiology and management of chronic hepatitis E infection in solid organ transplantation: A comprehensive literature review. Rev. Med. Virol..

[3] Bustamante N.D., Matyenyika S.R., Miller L.A., Goers M., Katjiuanjo P., Ndiitodino K., Ndevaetela E.E., Kaura U., Nyarko K.M., Kahuika-Crentsil L. (2020). Notes from the Field: Nationwide Hepatitis E Outbreak Concentrated in Informal Settlements–Namibia, 2017–2020. MMWR Morb. Mortal. Wkly. Rep..

[4] Wang B., Akanbi O.A., Harms D., Adesina O., Osundare F.A., Naidoo D., Deveaux I., Ogundiran O., Ugochukwu U., Mba N. (2018). A new hepatitis E virus genotype 2 strain identified from an outbreak in Nigeria, 2017. Virol. J..

[5] World Health Organization (WHO) Hepatitis E. https://www.who.int/news-room/fact-sheets/detail/hepatitis-e.

[6] Cong W., Sui J.C., Zhang X.Y., Qian A.D., Chen J., Zhu X.Q. (2015). Seroprevalence of hepatitis E virus among pregnant women and control subjects in China. J. Med. Virol..

[7] Dagnew M., Belachew A., Tiruneh M., Moges F. (2019). Hepatitis E virus infection among pregnant women in Africa: Systematic review and meta-analysis. BMC Infect. Dis..

[8] Mirzaev U.K., Ouoba S., Ko K., Phyo Z., Chhoung C., Ataa A.G., Sugiyama A., Akita T., Tanaka J. (2024). Systematic review and meta-analysis of hepatitis E seroprevalence in Southeast Asia: A comprehensive assessment of epidemiological patterns. BMC Infect. Dis..

[9] Ahmed M.K., Maroofi H., Blunt M., Labrique A., Kirkwood C., Vannice K., Talaat K.R., Lynch J., Kmush B.L. (2026). Current global estimates, risk factors, and knowledge gaps for Hepatitis E virus (HEV): A scoping review. PLoS Negl. Trop. Dis..

[10] Kogias D., Skeva A., Smyrlis A., Mourvati E., Kantartzi K., Romanidou G., Kalientzidou M., Rekari V., Konstantinidou E., Kiorteve P. (2023). Hepatitis E Virus (HEV) Infection in Hemodialysis Patients: A Multicenter Epidemiological Cohort Study in North-Eastern Greece. Pathogens.

[11] Kogias D., Kouklakis G., Papadopoulos V. (2026). Hepatitis E Infection in Patients with Inflammatory Bowel Diseases: A Systematic Review and Meta-Analysis. J. Viral Hepat..

[12] Hakim M.S., Wang W., Bramer W.M., Geng J., Huang F., de Man R.A., Peppelenbosch M.P., Pan Q. (2017). The global burden of hepatitis E outbreaks: A systematic review. Liver Int..

[13] Patra S., Kumar A., Trivedi S.S., Puri M., Sarin S.K. (2007). Maternal and fetal outcomes in pregnant women with acute hepatitis E virus infection. Ann. Intern. Med..

[14] Navaneethan U., Al Mohajer M., Shata M.T. (2008). Hepatitis E and pregnancy: Understanding the pathogenesis. Liver Int..

[15] Tosone G., Simeone D., Spera A.M., Viceconte G., Bianco V., Orlando R. (2018). Epidemiology and pathogenesis of fulminant viral hepatitis in pregnant women. Minerva Ginecol..

[16] Rezig D., Ouneissa R., Mhiri L., Mejri S., Haddad-Boubaker S., Ben Alaya N., Triki H. (2008). Séroprévalences des infections à hépatite A et E en Tunisie [Seroprevalences of hepatitis A and E infections in Tunisia]. Pathol. Biol..

[17] Hannachi N., Boughammoura L., Marzouk M., Tfifha M., Khlif A., Soussi S., Skouri H., Boukadida J. (2011). Le risque infectieux viral chez le polytransfusé: Séroprévalence de sept agents viraux dans le centre tunisien. Bull. Soc. Pathol. Exot..

[18] Hellara O., Melki W., Mastouri M., Chaabène N.B., Loghmari H., Mansour W.B., Bdioui F., Safer L., Saffar H. (2014). Sérodiagnostic des hépatites aiguës de l’adulte: Résultats d’une étude prospective dans une région du centre tunisien. Tunis. Med..

[19] Ben-Ayed Y., Hannachi H., Ben-Alaya-Bouafif N., Gouider E., Triki H., Bahri O. (2015). Hepatitis E virus seroprevalence among hemodialysis and hemophiliac patients in Tunisia (North Africa). J. Med. Virol..

[20] Houcine N., Jacques R., Salma F., Anne-Gaelle D., Amin S., Mohsen H., Hamadi B., Christophe R., Patrice A., Mahjoub A. (2012). Seroprevalence of hepatitis E virus infection in rural and urban populations, Tunisia. Clin. Microbiol. Infect..

[21] Béji-Hamza A., Hassine-Zaafrane M., Khélifi-Gharbi H., Della Libera S., Iaconelli M., Muscillo M., Petricca S., Ciccaglione A.R., Bruni R., Taffon S. (2015). Hepatitis E virus genotypes 1 and 3 in wastewater samples in Tunisia. Arch. Virol..

[22] Neffatti H., Lebraud P., Hottelet C., Gharbi J., Challouf T., Roque-Afonso A.M. (2017). Southern Tunisia: A still high endemicity area for hepatitis A. PLoS ONE.

[23] Hannachi N., Hidar S., Harrabi I., Mhalla S., Marzouk M., Ghzel H., Ghannem H., Khairi H., Boukadida J. (2011). Séroprévalence et facteurs de risque de l’hépatite virale E chez la femme enceinte dans le centre tunisien [Seroprevalence and risk factors of hepatitis E among pregnant women in central Tunisia]. Pathol. Biol..

[24] Costa M.L., Souza R.T., Cecatti J.G., Gottlieb S.L., Delnord M., Thwin S.S., Habib N.A., Silva R., Giordano D., Thorson A. (2025). Implementation of the multicountry WHO COVID-19 pregnancy cohort study: Challenges and lessons learned during the pandemic. Reprod. Health.

[25] Jothikumar N., Cromeans T.L., Robertson B.H., Meng X.J., Hill V.R. (2006). A broadly reactive one-step real-time RT-PCR assay for rapid and sensitive detection of hepatitis E virus. J. Virol. Methods.

[26] Inoue J., Takahashi M., Yazaki Y., Tsuda F., Okamoto H. (2006). Development and validation of an improved RT-PCR assay with nested universal primers for detection of hepatitis E virus strains with significant sequence divergence. J. Virol. Methods.

[27] La Rosa G., Fratini M., Muscillo M., Iaconelli M., Taffon S., Equestre M., Chionne P., Madonna E., Pisani G., Bruni R. (2014). Molecular characterisation of human hepatitis E virus from Italy: Comparative analysis of five reverse transcription-PCR assays. Virol. J..

[28] Li P., Liu J., Li Y., Su J., Ma Z., Bramer W.M., Cao W., de Man R.A., Peppelenbosch M.P., Pan Q. (2020). The global epidemiology of hepatitis E virus infection: A systematic review and meta-analysis. Liver Int..

[29] La Fauci V., Facciolà A., Riso R., Calimeri S., Lo Giudice D., Squeri R. (2017). Seroprevalence of hev antibodies in a sample of pregnant women in the city of Messina. Ann. Ig..

[30] Farshadpour F., Taherkhani R., Ravanbod M.R., Eghbali S.S., Taherkhani S., Mahdavi E. (2018). Prevalence, risk factors and molecular evaluation of hepatitis E virus infection among pregnant women resident in the northern shores of Persian Gulf, Iran. PLoS ONE.

[31] Ma X.X., Ji Y., Jin L., Baloch Z., Zhang D.R., Wang Y., Pan Q., Ma Z. (2021). Prevalence and clinical features of hepatitis E virus infection in pregnant women: A large cohort study in Inner Mongolia, China. Clin. Res. Hepatol. Gastroenterol..

[32] Noufensi M.R.Y., Kengne M., Njukeng P.A., Anong D.N., Masebe T.M., Tamoufe U., Echelibe H., Ter Goon D., Nwobegahay J.M. (2016). Seroprevalence and risk factors of hepatitis E virus infection among pregnant women at the Yaounde central hospital, Cameroon. Microbiol. Res. Int..

[33] Alkali B.R., Bello M., Kabiru M., Shu’aibu A.B., A’isha B.I., Firdausi A., Bunza N.M., Ashcroft O.F. (2016). Seroprevalence of hepatitis E virus (HEV) infection in pregnant women in Sokoto state, Nigeria. J. Adv. Microbiol..

[34] Eltayeb R., Gasim I.G., Elhassan M.E., Abdullahi H., ARayis D. (2015). Maternal and newborn Seroprevalence of hepatitis E virus at Medani Hospital, Sudan. F1000Research.

[35] Caron M., Kazanji M. (2008). Hepatitis E virus is highly prevalent among pregnant women in Gabon, central Africa, with different patterns between rural and urban areas. Virol. J..

[36] Gad Y.Z., Mousa N., Shams M., Elewa A. (2011). Seroprevalence of subclinical HEV infection in asymptomatic, apparently healthy, pregnant women in Dakahlya Governorate, Egypt. Asian J. Transfus. Sci..

[37] Boonyai A., Thongput A., Sisaeng T., Phumchan P., Horthongkham N., Kantakamalakul W., Chaimayo C. (2021). Prevalence and clinical correlation of hepatitis E virus antibody in the patients’ serum samples from a tertiary care hospital in Thailand during 2015–2018. Virol. J..

[38] Avellon A., Morago L., Garcia-Galera del Carmen M., Munoz M., Echevarría J.M. (2015). Comparative sensitivity of commercial tests for hepatitis E genotype 3 virus antibody detection. J. Med. Virol..

[39] Candido A., Taffon S., Chionne P., Pisani G., Madonna E., Dettori S., Hamza A., Valdarchi C., Bruni R., Ciccaglione A.R. (2012). Diagnosis of HEV infection by serological and Real-time RT-PCR assays: A study on acute non-A-C hepatitis collected from 2004 to 2010 in Italy. BMC Res. Notes.

[40] Tissera G., Lardizabal M.C., Torres S.B., Fantilli A.C., Martínez Wassaf M.G., Venezuela F., Capra R., Balderramo D.C., Travella C., Ré V.E. (2020). Hepatitis E virus infection in pregnant women, Argentina. BMC Infect. Dis..

