# Supplementary figures and images for: Prevalence of Hepatitis E Virus Infection Among Pregnant Women in Tunisia: Findings from a Large Cohort Study
Source: Pathogens. 2026 May 19;15(5):549. doi: 10.3390/pathogens15050549 (PMC13209197; doi:10.3390/pathogens15050549)

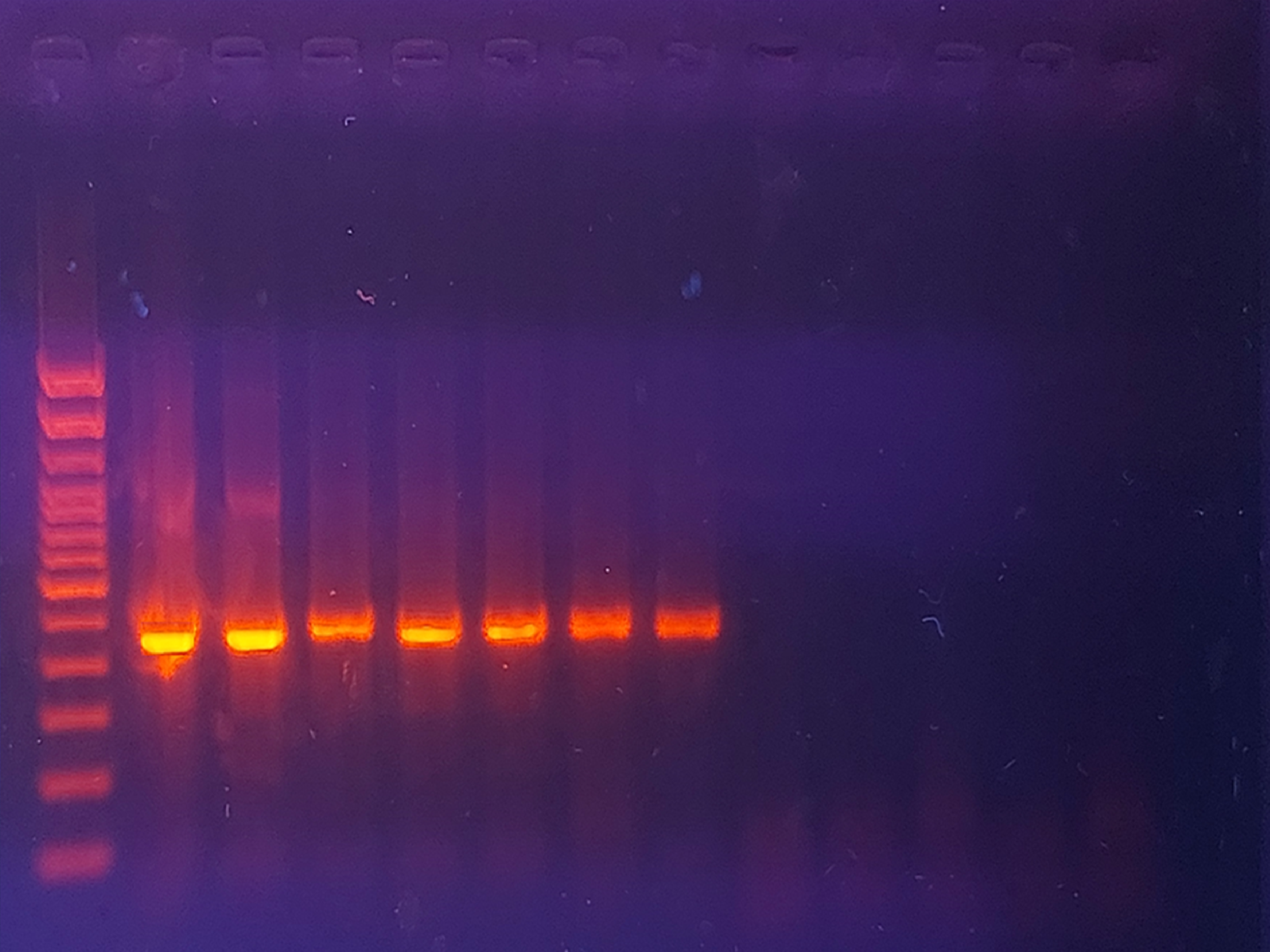

Supplement: Supplementary file 1 [file pathogens-15-00549-s001.zip › Figure S1.png]
